# Editorial: Advanced Analytics and Decision Making for Research Policy and Strategic Management

**DOI:** 10.3389/frma.2021.778622

**Published:** 2021-11-08

**Authors:** Yi Zhang, Yuya Kajikawa

**Affiliations:** ^1^ Australian Artificial Intelligence Institute, Faculty of Engineering and Information Technology, University of Technology Sydney, Ultimo, NSW, Australia; ^2^ Graduate School of Environment and Society, Tokyo Institute of Technology, Tokyo, Japan; ^3^ Institute for Future Initiatives, The University of Tokyo, Tokyo, Japan

**Keywords:** bibliometrics, data analytics, research policy, decision making, strategic management

New challenges are significantly raising for making decisions in real-world complicated environments, such as processing large-scale data, recognizing complex relationships, investigating hidden mechanisms, forecasting future trends, and developing models with robustness and adaptability. Bibliometrics, as an effective tool, has provided systematic solutions for analyzing bibliometric data (e.g., research articles, patents, academic proposals, news, and web pages), and thus supporting research policy and strategic management. Further, the rapid development of information technologies, particularly, artificial intelligence and data science techniques, brings evolutionary changes to traditional bibliometrics, as well as its broad applications. For example, large-scale data analytics facilitates citation networks and textual segments to identify emerging topics and discover latent connections among multiple bibliometric sources (Takano and Kajikawa, 2019; Mejia and Kajikawa, 2020; Mejia and Kajikawa, 2021), and network analytics leverage understandings on the topological structure of bibliometric networks to enable new angles for evaluating science, technology, and innovation (Zhang et al., 2021a, 2021b).

This topic aims to explore the ways of fully facilitating the power of advanced analytics to enhance its capability in decision support for research policy and strategic management in scalable, uncertain and complicated environments in the real world. Targeting to broad issues in science, technology and innovation (ST&I) studies, this topic emphasizes the development and applications of advanced analytic approaches that provide transparent, explainable, and evaluable evidence for decision making. The topic also aims to focus on roles of analytics in decision making processes. Despite recent advancement of analytical technologies, there are still unfulfilled gaps among data, evidence, decision support and decision making. We also seek effective organizational methods and protocols to promote adaptation of advanced analytics. This cutting-edge topic is to spearhead a cross-disciplinary direction crossing disciplines such as information science, computer science, and broad business disciplines. We expect the topic will create fundamental theories, conceptual methodologies, and practical toolkits for advancing studies and practices in research policy and strategic management.

## Contributions

This topic collects four manuscripts, touching diverse aspects of advanced analytics, modeling, and their applications for research policy and strategic management.

Aiming to improve the performance of profiling the demographic characteristics of learners in the platform of Massive Open Online Course (MOOCs), Aljohani and Cristea developed a novel solution by incorporating certain state-of-the-art deep learning models and architectures for text segmentation and analytics. Such demographic characteristics specifically focus on employment status and gender information.

While arguing the accessibility of traditional bibliometric data sources as well as nonspecific and outdated “global” measures, Hook and Porter facilitated the Dimensions database in the Google BigQuery Cloud environment and proposed a visualization solution for mapping institutions and their productivity in a geographical map.

With a specific interest in profiling COVID-19-related genetic research, Wu et al. proposed a solution exploiting a set of intelligent bibliometric models, such as scientific evolutionary pathways for tracking topic evolution and bio-entity network analytics for discovering gene-disease associations.

As a conclusion for this special topic, the topic editors and their colleagues particularly investigated topics and their evolutionary pathways in bibliometric research (Mejia et al.). In this study, the authors used several advanced bibliometric tools, such as citation network analytics, scientific evolutionary pathways, and hierarchical topic trees, to profile the keen interests of the bibliometric community and how such interests changes over time in the past decades.

With the four submissions in this collection, we observed that using, refining, and developing advanced analytic models, incorporating with state-of-the-art artificial intelligence and data science techniques, has become a rising trend in bibliometrics, and such a trend further adapts to actual needs from the community of research policy and strategic management, who are facing challenging issues on decision support in changing environments. Given that, we believe this special topic may feed readers with both novel methodological solutions and ingenious practical applications.
